# Estimating and modelling the transmissibility of Middle East Respiratory Syndrome CoronaVirus during the 2015 outbreak in the Republic of Korea

**DOI:** 10.1111/irv.12467

**Published:** 2017-08-17

**Authors:** Xu‐Sheng Zhang, Richard Pebody, Andre Charlett, Daniela de Angelis, Paul Birrell, Hunseok Kang, Marc Baguelin, Yoon Hong Choi

**Affiliations:** ^1^ National Infection Service Centre for Infectious Disease Surveillance and Control Public Health England London UK; ^2^ Department of Infectious Disease Epidemiology Medical Research Council Centre for Outbreak Analysis and Modelling Imperial College School of Public Health London UK; ^3^ Medical Research Council Biostatistics Unit University of Cambridge Cambridge UK; ^4^ Research Centre for Nonlinear Ergodic Theory Sungkyunkwan University Suwon Korea

**Keywords:** mathematical modelling, Middle Eastern Respiratory Syndrome CoronaVirus, parameter estimation, South Korean outbreak, statistical analysis, transmissibility

## Abstract

**Background:**

Emerging respiratory infections represent a significant public health threat. Because of their novelty, there are limited measures available to control their early spread. Learning from past outbreaks is important for future preparation. The Middle Eastern Respiratory Syndrome CoronaVirus (MERS‐CoV ) 2015 outbreak in the Republic of Korea (ROK) provides one such opportunity.

**Objectives:**

We demonstrated through quantitative methodologies how to estimate MERS‐CoV's transmissibility and identified the effective countermeasures that stopped its spread.

**Methods:**

Using the outbreak data, statistical methods were employed to estimate the basic reproductive number *R*_0_, the average number of secondary cases produced by a typical primary case during its entire infectious period in a fully susceptible population. A transmission dynamics model was also proposed to estimate *R*_0_ and to identify the most effective countermeasures. The consistency between results will provide cross‐validation of the approaches.

**Results:**

*R*_0_ ranged from 2.5 with 95% confidence interval (CI): [1.7, 3.1] (using the sequential Bayesian method) to 7.2 with 95% CI: [5.3, 9.4] (using the Nowcasting method). Estimates from transmission model were higher but overlapped with these. Personal protection and rapid confirmation of cases were identified as the most important countermeasures.

**Conclusions:**

Our estimates were in agreement with others from the ROK outbreak, albeit significantly higher than estimates based on other small outbreaks and sporadic cases of MERS‐CoV. The large‐scale outbreak in the ROK was jointly due to the high transmissibility in the healthcare‐associated setting and the Korean culture‐associated contact behaviour. Limiting such behaviour by rapidly identifying and isolating cases and avoiding high‐risk contacts effectively stopped further transmission.

## 1 INTRODUCTION

Quickly measuring transmissibility of an emerging respiratory infectious disease is vital to preparedness of authorities and design of the optimal intervention strategies. The key quantity that characterises the transmissibility is the basic reproductive number (*R*_0_), the average number of new infections caused by a single infective individual introduced into a completely susceptible population.^1^ It is a threshold parameter: if *R*_0_<1, the disease dies out without any intervention; otherwise, the disease can persist. To monitor how transmissibility evolves and whether the countermeasures can reduce the transmission along the outbreak course, the effective reproduction number (*R*_t_) is also a useful parameter. Following the 2003 SARS outbreak, especially the 2009 pandemic flu, effort has been made to rapidly estimate the reproductive number and many statistical methods^2^, ^3^, ^4^, ^5^, ^6^, ^7^ were proposed for this task. Fortunately, these methods have now been made in publicly available software.^8^, ^9^

Middle Eastern Respiratory Syndrome CoronaVirus (MERS‐CoV), a zoonotic virus, was first identified in Saudi Arabia in June 2012. Multiple introductions to the human population have occurred from the animal reservoir in the Middle East, with person‐to‐person transmission well documented in healthcare and household settings. Sporadic, exported cases have been reported in Europe, Africa, Asia and North America since, with cases linked with returning travel from the Middle East. Although limited transmission was reported in these other regions, no sustained onward transmission has been detected outside of the Middle East until 2015. MERS‐CoV transmissibility has been estimated to be around the threshold value of 1.0, ranging from 0.4 to 1.5.^10^, ^11^, ^12^ These estimates are mainly based on sporadic cases and self‐limited clusters.^13^ However, the estimation^14^ on a large healthcare‐associated outbreak in Jeddah and Riyan, the Kingdom of Saudi Arabia (KSA) during spring 2014, with over 300 cases, suggested a higher *R*_0_ ranging from 2.0 to 6.7.

The epidemic in the Republic of Korea (ROK) during 2015 was seeded from a traveller returned from Bahrain after visiting the United Arab Emirates and the KSA, and caused an outbreak of 185 confirmed cases. It has been the largest outbreak outside of the Middle East so far,^15^ and much theoretical attention has been attracted to estimate its transmissibility. Hsieh^16^ used a phenomenological model to obtain an estimate of *R*_0_ ranging from 7.0 to 19.3. Xia et al.^17^ and Kim et al.^18^ used transmission dynamics models and obtained *R*_0_ = 4.4 and 5.4, respectively. Reconstructing the transmission tree and considering the heterogeneity in the transmission processes, Nishiura et al.^19^ estimated that the reproductive number throughout the whole outbreak has a mean of about 1.0 and a variance of 52.1 (it is worth mentioning that the mean reproductive number throughout the outbreak course is of no help for understanding the transmissibility of the causing pathogens).* These estimates differ from the studies based on previous MERS‐CoV outbreaks^10^, ^11^, ^12^ and show a quite diverse picture of *R*_0_ for the ROK outbreak. In general, the transmissibility depends not only on the biological properties of a pathogen such as transmission mode and infectivity, but also on the susceptibility and contact patterns of the host populations.^1^ The difference in transmissibility among different locations and ethnic populations highlights the importance of its setting dependency. It implies that given the same infectivity of a pathogen, the size and duration of the outbreaks it causes will depend on the contact patterns of the population attributable to their culture‐associated behaviours or geographical‐related environment. Different from Westernised culture, Koreans have the tradition to visit relatives and friends in hospitals and can choose the hospitals that they think are the best for their treatment. This culture‐associated behaviour along with increased infectivity in hospital environment^10^, ^20^ may have facilitated the spread of MERS‐CoV in the ROK.^21^

Both Xia et al.^17^ and Kim et al.^18^ used dynamics models to identify the main determinants of transmission in the ROK outbreak. Both assume that the hospitalised patients can transmit infection to others, which is not true in the ROK outbreak where no infection was caused by confirmed cases as they were all isolated after the confirmation.^21^ Further, they both fixed the transition rates so that the stage durations (e.g incubation period and delay from symptom onset to hospitals) were implicitly assumed to be exponential. This is also not true as the observed stage durations are non‐exponentially distributed (see Appendix S1). They used the least‐square methods to estimate the model parameters, which, in view of a huge amount of uncertainty and heterogeneity in the outbreak,^19^ may not be appropriate. To comprehensively understand how these variations affect the estimation of transmissibility, a more general methodology such as Bayesian inference is needed which combined case data and the priors extracted from previous studies or direct estimates from the outbreak data.

In this study, we revisit the estimation of reproductive number of MERS‐CoV using statistical methods on the outbreak data released by Korea Centers for Disease Control and Prevention (KCDC)^22^ Furthermore, by considering the actual situation of transmission events, we propose a transmission dynamics model to explore how the variation in transition rates affects the decomposition of the key pathways of the spread. The dynamic modelling aims to shed useful insights into the design of effective intervention strategies, which will be critical for controlling emerging respiratory outbreaks in future.

## 2 DATA

The ROK outbreak started from one traveller from the Middle East who was confirmed with the MERS‐CoV on 20 May 2015. This outbreak resulted in 186 cases including 38 deaths. Cases were scattered across the country. One case travelled to China, was confirmed and treated there; under the Chinese government's rapid response and control programme, the case did not cause any onward transmission. Detailed outbreak data are available from KCDC.^22^ The information on each confirmed case included symptom‐onset date, confirmation date, infection place, possible infectors, first and last exposure dates and date of recovery or death.

All the 186 cases had confirmation dates, but only 179 cases had symptom‐onset dates with three healthcare workers who tested positive reportedly having no symptoms and another four cases reportedly having symptoms but no symptom‐onset dates reported. To use all the 186 cases, we regarded asymptomatic cases as symptomatic, and imputed the symptom‐onset dates as following: for each of the seven cases, any date before its confirmation date could be its illness onset date with probabilities given by a gamma distribution obtained from the delays from symptom onset to confirmation of the 179 cases. Potential exposure windows for 184 cases were recorded. We generated exposure dates by assuming the actual dates were uniformly distributed over the exposure windows for each case. For other two cases that have no recorded exposure windows but have symptom‐onset dates, any date before the symptom‐onset date could be the exposure date with probabilities given by the fitted incubation period distribution.

For 162 cases, the outbreak investigators observed a unique likely infector. As in the KSA nosocomial outbreaks,^23^ superspreaders were detected in the ROK outbreak: with only five cases responsible for the majority of confirmed cases and producing more than 80% of all 185 onward transmissions.

**Figure 1:**
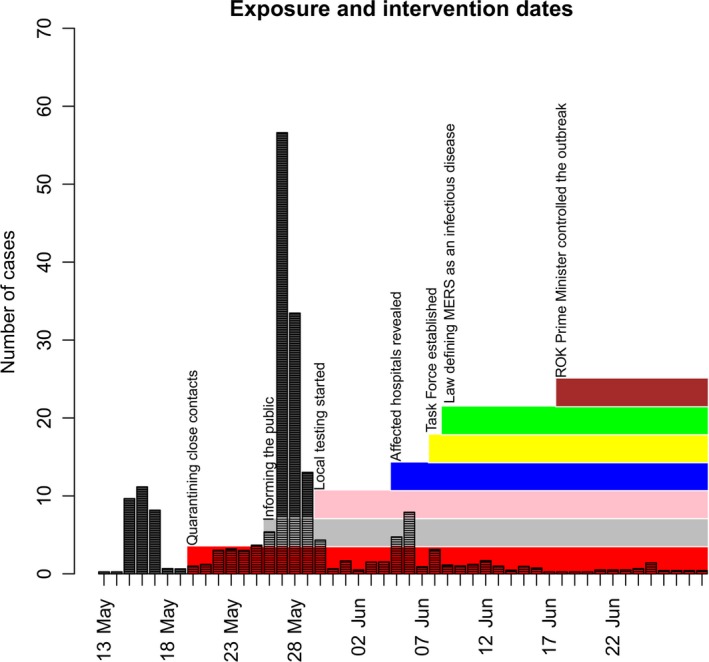
The timeline of intervention measures along with the exposure dates of cases. Here, exposure dates of cases are assumed to be uniformly distributed over the recorded potential exposure windows. The index case is exclusive with his exposure window from 29 April to 2 May 2015

The Central MERS‐CoV Control Office in Korea (KoMCO) informed daily on all new confirmed cases and their details from the fourth confirmed case on 26th May onwards through its websites (mainly in Korean and some in English) and the International Health Regulations (IHR) group on the World Health Organization (WHO) web site (in English). They started quarantining close contacts with confirmed patients from 20th May including family members and hospital staff. Prior to 30th May, only KCDC conducted the confirmation tests. From then, the tests were conducted in local provincial trusts. From 6th June, the government started revealing all hospitals where confirmed patients visited. The Task Force to control the outbreak was established on 8th June and extra 250 officers were put to deal with quarantining and the contact tracing work. On 18th June, the Prime Minister of the ROK announced that he would control the outbreak. There was no law defining MERS as a notifiable infectious disease until 9th June. The timeline of these intervention measures is shown in Figure 1.

## 3 METHODS AND MODELS

### 3.1 Modelling the transmissibility

To describe the ROK outbreak, we propose a transmission dynamics model by ignoring complexes such as age and geographical heterogeneity in transmission rate. MERS‐CoV is zoonotic and can transmit via direct contact or large virus‐laden droplets. It can pass from animals to humans and from humans to humans.^12^ Because of no zoonotic infection in the ROK outbreak, we only consider the human‐to‐human transmission. Although all transmissions occurred within hospitals, the affected hospitals were distributed across the ROK^21^, ^22^ so we assume the ROK population of size N=51 413 925 in mid‐2015^24^ are involved in the outbreak. On 4th May, all the people are assumed to be susceptible (*S*) except the index case who carried the virus but was not ill until 11th May. Contacts with cases will first become infected (*E*) and become infectious (*I*) after the latent period. In this study, we assume that latent period is equal to incubation. It has been noted elsewhere that asymptomatic MERS‐CoV infections are not rare^23^, ^25^; however, only three asymptomatic infections were detected among 16,752 close contacts during the ROK outbreak.^26^ As these asymptomatic cases do not cause further infections, we assume that all infected people are symptomatic and admitted to hospitals and then being confirmed (*C*). It is worth mentioning that during the ROK outbreak, all transmissions occurred in hospitals. That is, the people who got infected (except the index case) are patients in hospitals due to other diseases or healthcare workers or visitors who visited friends and relatives there. Once confirmed, the cases would be put under security and isolation in designated facilities. The ROK outbreak data show that no infection was caused by confirmed cases so we assume confirmed infections do not contribute to the transmission process.^17^, ^18^ Confirmed cases either recover or die. The ROK population is therefore decomposed into four compartments: S‐E‐I‐C and transmission dynamics is approximated by

$$\frac{dS}{dt}=-\omega \left(t\right)\beta \frac{I(t)S(t)}{N}$$

(1) $$\frac{dE}{dt}=\omega \left(t\right)\beta \frac{I(t)S(t)}{N}-E/L$$

$$\frac{dI}{dt}=\left(E/L\right)-\left(I/D\right)$$

$$\frac{dC}{dt}=I/D$$

The definitions and priors of parameters are listed in Table 1. We assume the epidemic starts from 11th May when the index case symptom onset with the initial seeding as: *E*(0) = 0, *I*(0) = 1, *C*(0) = 0. Two types of parameters are defined to reflect the people's response and countermeasures to the MERS‐CoV infection: the self‐protection coefficient (*ω*) in these who are infectious and before confirmation, and the delay from symptom onset to confirmation (*D*) reflecting the diagnosis testing rate. They are assumed to vary as,

$$\omega \left(t\right)=\left\{\begin{array}{cc} 1 & t<t_{1}\\ \omega & t\geq t_{1} \end{array}\right.$$

(2) $$D\left(t\right)\equiv \left\{\begin{array}{cc} D_{0} & t<t_{1}\\ D_{1} & t\geq t_{1} \end{array}\right.$$

with *t*_1_ representing the breaking point. As shown in Figure 1, no one measure event can assume significantly as a clearly subjective breaking point; *t*_1_ will be sought objectively by comparing the deviance information criterion (DIC) among model variants with different dates. The basic reproductive number before intervention at day *t*_1_ is,

(3a) $$R_{0}=\beta E\left(D_{0}\right)$$

and after the intervention, it becomes

(3b) $$R_{c}=\omega \beta E\left(D_{1}\right)$$

**Table 1:** Model parameters: their priors and posteriors

Parameters	Definition	Priors	Source	Posteriors
β_I_	Transmission coefficient	Γ(1.5, 2.0) with mean = 0.75 SD = 0.61	17	0.99 (95% CI: 0.74, 1.42)
*L*	Incubation period	Γ(4.44, 0.55) with mean = 8.07 SD = 3.83	Estimated (Figure A1 in Appendix S1)	8.19 (95% CI: 5.49, 11.66)
*D*_0_	Delay from symptom onset to confirmation before 28th May	Γ(3.28, 0.48) with mean = 6.83 SD = 3.77	Estimated (Figure A2 in Appendix S1)	9.26 (95% CI: 4.25, 18.03)
*D*_1_	Delay from symptom onset to confirmation after 28th May	Γ(3.28, 0.48) with mean = 6.83 SD = 3.77	Estimated (Figure A2 in Appendix S1)	4.05 (95% CI: 1.80, 6.93)
ω	Self‐protection coefficient	Γ(2, 2) with mean = 0.50, SD = 0.22	17	0.091 (95% CI: 0.043, 0.235)
η	Dispersion parameter	Γ(3.125, 0.3125) with mean = 10.0, SD = 5.6	29	3.72 (95% CI: 2.89, 4.93)
	Serial interval	Γ(9.83, 0.72) with mean = 13.65, SD = 4.35	Estimated (Figure A3 in Appendix S1)	aΓ(8.33, 0.66) mean = 12.62 days SD = 4.37 days
*R*_0_	Basic reproductive number	–	–	9.11 (95% CI: 5.32, 15.92)
*R*_c_	Reproductive number after effective intervention	–	–	0.368 (95% CI: 0.251, 0.508)

a Estimate from transmission tree reconstructed by the method of Hens et al.^7^

Here, E() stands for the mean of the distribution. To test model sensitivity to variation in people's responses, we also consider the simplified situation by ignoring the differences in people's response and assuming a same diagnosis rate (i.e a constant delay from symptom onset to confirmation) over the whole outbreak.

Here, it is worth discussing the target population of transmission dynamics of MERS‐CoV infection in the ROK. All cases in the outbreak, including healthcare workers, patients and visitors, were linked to healthcare settings^19^, ^27^; it is thus appropriate to assume that the transmission only acts on the people in the healthcare facilities as did in Lee et al.^28^ However, it should be noticed that the people in the ROK can freely visit any hospitals they want and relatives and friends have the tradition to visit the patients in hospitals.^21^ This may indicate that the infection can actually spread on a wide and large population. Technically, as the frequency‐dependent contact rate in equation (2) was assumed, whether using N = 51 413 925 or N = 10 000 as,^28^ provided N≫186, this will not affect the estimation of model parameters.

#### 3.1.1 Inference model

To reflect the huge dispersion in the daily number of cases, the negative binomial likelihood function was used. The number *x* ^*i*^(*t*) of cases on day *t* is distributed as

(4) $$l^{i}\left(x^{i}\left(t\right)|\Theta ,\eta \right)=\frac{\Gamma (x^{i}\left(t\right)+r_{t}^{i})}{\Gamma \left(r_{t}^{i}\right)\Gamma \left(x^{i}\left(t\right)+1\right)}{\left(\frac{1}{\eta }\right)}^{r_{t}^{i}}{\left(1-\frac{1}{\eta }\right)}^{x^{i}\left(t\right)},$$

where

(5) $$r_{t}^{i}=\mu {}^{i}\left(t\right)/\left(\eta -1\right)$$

Here, η is the dispersion parameter and μ^*i*^(*t*) are the predictions of the cases on day *t* from the transmission dynamics. Here, index *i* stands for three different observational data: infection (E), symptomatic cases (I) and confirmed cases (C).

Assuming that the observed incidence *x* ^*i*^(1), *x* ^*i*^(2),…, *x* ^*i*^(*T* ^*i*^) are conditionally independent, the total likelihood given parameters **Θ** is

(6) $$L\left(\Theta ,\eta ;x\right)=\prod_{i=E,I,C}\prod_{\tau =t^{i}}^{T^{i}}l^{i}\left(x^{i}\left(\tau \right)\right|\Theta ,\eta ),$$

where the starting points of the three series are *t* ^E^ = 3, *t* ^I^ = 1, *t* ^C^ = 10, respectively, and their end points are *T* ^E^ = 48, *T* ^I^ = 53, *T* ^C^ = 55. (As the exposure date of the index case is earlier than 11th May, only 185 cases are used for the exposure date series.)

The priors *f*(**Θ**) for parameters are extracted from the literature or direct estimation from the ROK outbreak data (see Table 1). Employing Bayesian framework through the combination of the priors *f*(**Θ**) and the likelihood *L*(**Θ**,η*;**x***), the posterior distribution can be obtained by Markov chain Monte Carlo simulations (MCMC). From these samples, we obtain means and their 95% confidence intervals (CIs) for parameters.^29^

The DIC that was used to compare the performance of model variants is defined^30^ as

(7) $$\text{DIC}=\frac{1}{2}\text{Var}\left(\text{Dev}(\Theta ,\eta )\right)-2\text{log}\left(L(\Theta ,\eta ;x)\right)$$

The most parsimonious model variant is the one that has the smallest DIC.

### 3.2 Estimating the transmissibility

Some packages coded in R‐computing language are available for estimating the transmissibility once the incidence time series data and serial interval (SI), which is defined as the difference in symptom‐onset dates of infectee‐infector pairs, are known. The six approaches used are listed in Table 2 and briefly described below. For these methods, we assume the SI distribution of mean = 12.62 days and SD = 4.29 days, which is directly estimated from the data (Figure A3 in Appendix S1) and close to the previous estimate by Cowling et al.^31^

**Table 2:** Comparison of statistical methods used for estimating *R*_0_ of MERS‐CoV in the ROK outbreak

Method	EG	ML	SB	EpiEstim	TD	Transmission tree
Reference	3	6	5	4, 9	2	7
Data required	Incidence data during the early phase of an outbreak; GT	Incidence data during early phase of exponential growth; GT	Incidence data during the early phase of an outbreak; infectious period	Incidence data; GT	Incidence data (symptom‐onset dates); GT	Incidence data (symptom‐onset dates); contact information
Mixing required	No	No	Random	Random	Random	No
Output	Exponential growth rate and the best *R*_0_ selected by deviance‐based *r*‐squared statistic	The best *R*_0_ selected by deviance *r*‐squared measure	*R*_0_ over the period of exponential growth	Effective reproductive number (*R*_*t*_) averaging over a fixing window	Symptom onset‐based transmission tree and *R*_*t*_ by averaging overall all transmission networks	Transmission tree, *R*_*t*_ and serial interval distribution
Results	6.36 [4.25, 9.68] Exponential growth rate: 0.155 [0.119, 0.194] See Appendix S1	5.89 [4.42, 7.66] See Appendix S1	2.45 [1.68, 3.12] See Appendix S1	7.15 [5.26, 9.35] See Figure 3	5.19 [3.34, 7.45] See Figure 4A	6.18 [2.80, 14.6] See Figure 4B

EG, exponential growth rate method; ML, maximum‐likelihood method; SB, sequential Bayesian method; TD, time‐dependent transmission tree method using tree reconstruction method.^2^ The four methods are coded in package “R0.”^8^

GT: generation time, time gap in infected times between an infectee and its infector which is usually approximated by serial interval—the gap in symptom onset between an infectee and its infector.

*Exponential growth rate method* 3 estimates *R*_0_ by formula *R*_0_ = 1/M(−*r*), where *r* denotes the estimate of initial exponential growth rate and M stands for the moment generating function of SI distribution; *maximum‐likelihood estimation method* 6 assumes that the number of secondary cases caused by an index case is Poisson‐distributed with expected value *R*_0_; *sequential Bayesian method* 5 approximates the SIR model by assuming the incidence at next time point is Poisson‐distributed with mean equalling to the product of the current incidence and exp{(*R*_0_ − 1)/(infectious period)}; *time‐dependent transmission tree method* 2 estimates reproduction numbers using transmission tree based only on the gaps in symptom‐onset dates between patients. These four methods are coded in the R0 package.^8^

Package “EpiEstim” ,^9^ which was based on the Nowcasting method^4^ that takes censoring into account, provides a method to estimate the instantaneous reproductive number (*R*_t_) smoothing by averaging over an interval. The transmission tree method^7^ is a development of another method^2^ by further combining the possible contact information to construct the transmission tree. The new method applies Bayesian inference to simultaneously estimate the most likely transmission tree and SI distribution.

## 4 RESULTS

### 4.1 Model selection of transmission dynamics

**Figure 2:**
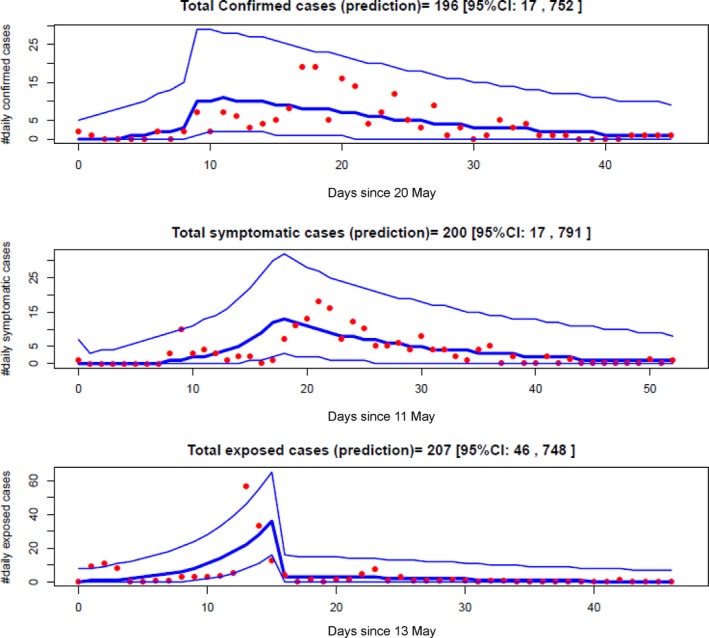
Transmission dynamics model fitting to the confirmed, symptomatic and exposed cases data under model assuming the breaking point at 28th May in both contact and diagnosis rates. Red filled circles are the cases data, thick blue lines represent the median predictions from transmission dynamics model, and the thin blue lines represent 95% credible intervals

The model comparison (Table 3) shows that the model variants with the breaking point in contact rate and diagnosis response to infection at 28 May 2015 are the best. Figure 2 illustrates the model fitting with data for the best model variant. In accordance with the suggestion of Spiegelhalter et al.,^32^ however, model variants with the breaking point at 29th May are not substantially different from the best variant, and are also good. This suggests that the effectively breaking point took place on 28th or 29th May. This is in agreement with the result of Hsieh^16^ who fitted the Richards model to cumulative case data. Transmission dynamics modelling suggests the reproductive number before the intervention (*R*_0_) has median 9.11 and 95% CI [5.32, 15.92] with the breaking point at 28th May (or 6.85 [4.13, 11.92] if the breaking point is at 29th May) for the model assuming both contact rate and diagnosis rate vary with the breaking point (Table 1 and Table C1 in Appendix S3). For the model that assumes only contact rate varies with the breaking point, the estimates of *R*_0_ are: 6.07 [4.20, 8.70] (5.36 [3.76, 7.60]) (see Tables C2 and C3 in Appendix S3). These estimates are compatible with the previous studies.^16^, ^17^, ^18^ Although all^16^, ^17^, ^18^ used cumulative data while we use daily incidence data, the similar estimates were obtained. This implies the limited influence of using different data set on the results. These estimates show that *R*_0_ of MERS‐CoV in the ROK is far beyond the threshold level.

**Table 3:** Comparison of model variants against the varying breaking points. Best DIC in bold

Model assumption	Both contact and diagnosis rates vary with the breaking point *t*_1_		Only contact rate varies with the breaking point *t*_1_	
Breaking point (*t*_1_)	DIC	*R*_0_ [95% CI]	DIC	*R*_0_ [95% CI]
25 May 2015	671.4	6.97 [3.64, 13.67]	680.0	3.46 [2.08, 5.98]
26 May 2015	656.9	8.07 [4.32, 15.32]	660.81	5.07 [3.05, 8.25]
27 May 2015	636.2	9.86 [5.56, 17.81]	639.4	6.20 [4.10, 9.24]
**28 May 2015**	**626.5**	**9.11 [5.32, 15.92]**	**628.2**	**6.07 [4.20, 8.70]**
29 May 2015	631.5	6.85 [4.13, 11.92]	630.8	5.36 [3.76, 7.60]
30 May 2015	644.5	5.65 [3.46, 9.74]	643.9	4.35 [3.02, 6.19]
31 May 2015	652.7	4.34 [2.76, 7.31]	650.7	3.70 [2.57, 5.26]
2 June 2015	669.8	2.99 [1.98, 4.86]	666.4	2.60 [1.81, 3.71]
4 June 2015	677.1	2.44 [1.64, 3.82]	673.1	2.15 [1.54, 3.01]
6 June 2015	688.0	1.78 [1.32, 2.64]	683.9	1.85 [1.34, 2.56]
8 June 2015	698.6	1.39 [1.17, 1.88]	701.7	1.51 [1.17, 2.10]

### 4.2 Statistical estimation of transmissibility

The three estimation methods (exponential growth rate, maximum likelihood and sequential Bayesian) show estimates of *R*_0_ (Table 2 and Appendix S2) with medians and 95% (CIs): 6.4 [4.3, 9.7], 5.9 [4.4, 7.7] and 2.5 [1.7, 3.1], respectively, which are consistently beyond the threshold level.

**Figure 3:**
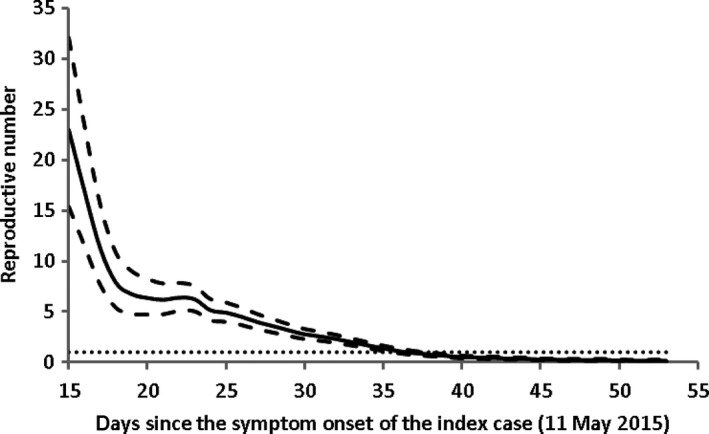
The effective reproductive number obtained by epiestim package. The estimates are obtained over a gap of 13 days. The symptom‐onset data are used for model fitting. Solid line represents the mean and dashed the upper and lower levels of 95% CIs. The horizontal dotted line represents the threshold value *R* = 1. The estimates show that *R*_t_ reduces to below 1.0 from 14th June (day 37)

The effective reproductive numbers (*R*_t_) are obtained from averaging over an interval^4^ and from construction of transmission tree.^2^, ^7^ The results from epiestim package^9^ slightly depends on the length of averaging interval (Figure 3). For example, with averaging interval = 13, 15 and 20 days, the *R*_t_ at day 23 (2nd June) has median and 95% CI as 6.23 [5.04, 7.54], 6.38 [5.18, 7.70] and 6.36 [5.16, 7.68], respectively. As 10th June (day 31) is a day with a delay of mean serial interval 13 days from the breaking point (28th May), in view of Nowcasting method^4^ that takes censoring into account, we regard the average of *R*_t_ before this date as an estimate of *R*_0_. Taking the averaging interval of 13 days, the median and 95% CI of *R*_0_ are 7.15 [5.26, 9.35].

**Figure 4:**
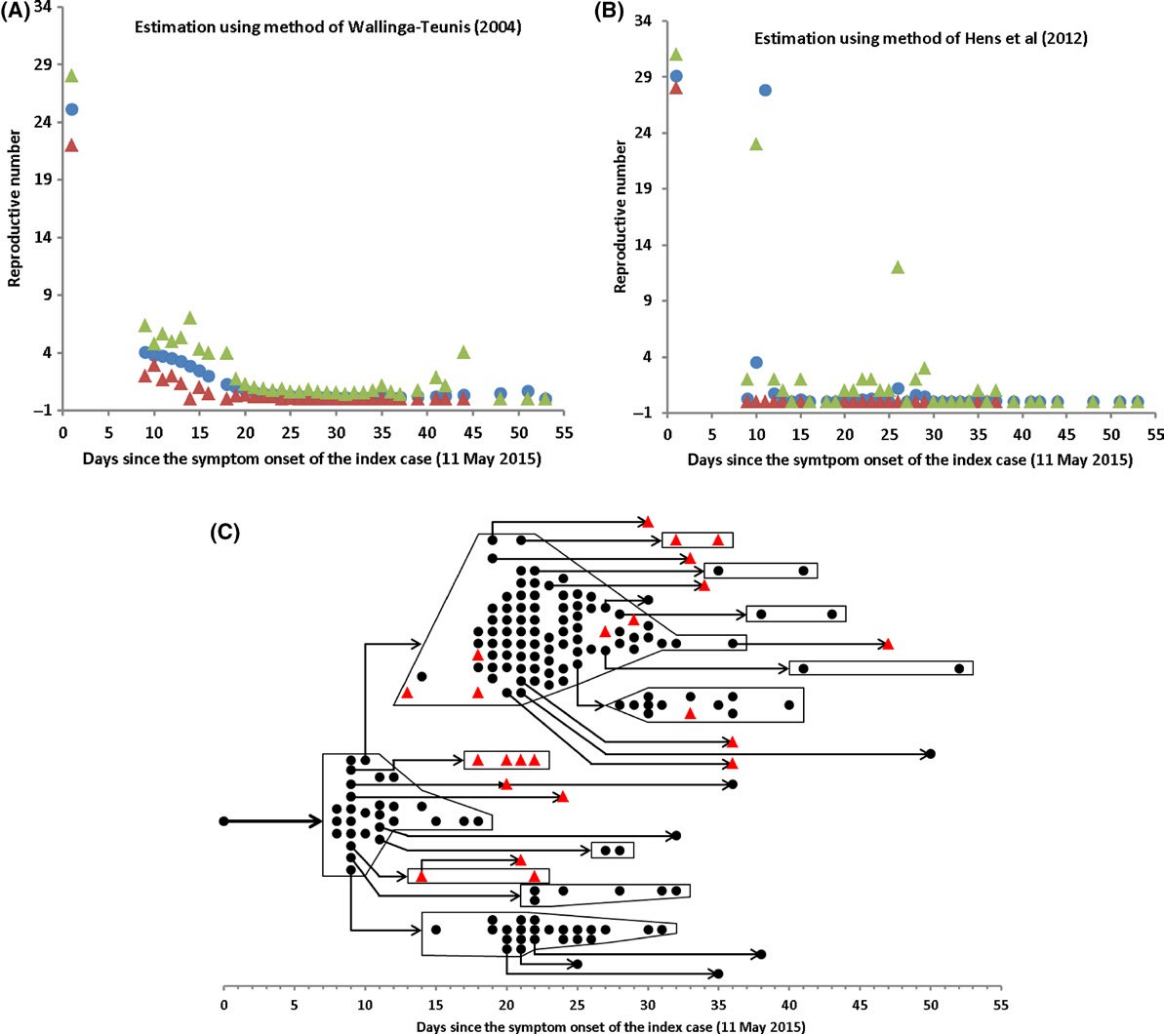
Transmission tree reconstruction and estimation of effective reproductive number. (A) Effective reproductive number (*R*_t_) estimated by the method^2^; (B) *R*_t_ by method.^7^ (C) A sample transmission tree reconstructed by method.^7^ In panels (A) and (B), filled circles represent means and triangles the lower and upper levels of 95% CIs. Notice the huge variation in Figure 4B, especially on day 11 (21st May), the *R*_t_ has mean 27.8 and 95% CI ranging from 0 to 85. (The 97.5% level point 85 is not shown in the Figure 4B.) In the transmission tree that describes who acquired infection from whom among 185 cases, 162 cases (black circles except index case) know their unique infectors and the infectors of other 23 cases (red triangles) were reconstructed by method^7^

Estimates of *R*_t_ from transmission trees reconstructed are shown in Figure 4. The two methods^2^, ^7^ show different patterns in *R*_*t*_ along the outbreak course: the relative smooth changes for the former (Figure 4A) and erratic evolution for the latter (Figure 4B). The huge variance in Figure 4B indicates the cluster transmissions. For example, three cases have symptom onset on 21st May; the estimate of *R*_*t*_ on the day has mean of 28 but a wide 95% CI ranging from 0 to 85. The large variation in *R*_*t*_ can also be seen on 20th May and 5th June. The difference between the two methods is because Hens et al.^7^ further include the information of infector‐infectee pair contacts. Nonetheless, the overall average decline patterns of *R*_*t*_ in Figure 4 appear similar to those shown in Figure 3. With the fully reconstructed transmission tree, we can easily estimate generation‐based reproductive number *R*_*g*_. From the sample transmission tree listed in Figure 4, we found *R*_*g*_ reduced quickly, from initially *R*_*g*_ = 28 to *R*_g_ = 4.43 with a large standard deviation of 16.2 on the second generation and on the third generation the mean of *R*_g_ became below the threshold level: *R*_g_ = 0.27 with a moderate standard deviation of 1.16. Approximating *R*_0_ by averaging *R*_*t*_ before 28th May (the breaking point), the mean and 95% CI are 5.2 [3.3, 7.5] and 6.2 [2.8, 14.6] by the methods of Wallinga and Teunis^2^ and Hens et al.^7^ respectively. The two methods obtained the similar mean but quite different variations. The reason is that Wallinga and Teunis^2^ implicitly assumed that the population is well mixing and that the probability of one case being an infector of another cases is only determined by the gap in symptom‐onset dates between the two cases. This difference also reflects the superspread: most infections are caused by a few of infections, while many other infections cause none.

### 4.3 Interventions

The transmission dynamics model considers the actual observation that the infected people can transmit infection to others only during the period from symptom onset to confirmation. Correlation analysis (Table 4) shows that the rapid diagnosis through shortening delay from symptom onset to confirmation and self‐protection are the main contributing factors to transmissibility. With countermeasures from 28th May, which reflect in both the self‐protection coefficient (*w* = 9%) and shortened delay from symptom onset to confirmation (from *D*_0_ = 9.3 days to *D*_1_ = 4.1 days; cf.,^33^), *R*_0_ is substantially reduced from 9.1 [5.3, 15.9] to below the threshold level: *R*_c_ = 0.37 [0.25, 0.51] so the transmission is completely under control and stops. The intervention is characterised by the increased quarantine in designated hospitals and self‐protection of the public to reduce the contact rate and the quick response to symptom onset for confirmation test with implementation of appropriate isolation procedures.

**Table 4:** Correlation coefficients with reproductive numbers under the best model variant. Highest correlation in bold

Input parameters	Correlation coefficient with	
	*R*_0_	*R*_c_
Transmission rate (β)	−0.313	−0.266
Incubation (*L*)	0.308	−0.424
Self‐protection (ω) from 28th May	−0.171	**0.650**
Delay from symptom onset to confirmation (*D*_0_) before 28th May	**0.911**	−0.166
Delay from symptom onset to confirmation (*D*_1_) after 28th May	0.167	−0.243
Dispersion parameter (η)	0.0953	0.306

## 5 DISCUSSION

The characterisations based on the previous small outbreaks and sporadic cases suggest that MERS‐CoV is severe but not very contagious.^10^, ^11^, ^12^ The ROK outbreak caused 185 new cases and lasted about 2 months (Figures 1 and 4C). All 186 cases were put into special care and 38 died in the end. In this study, we applied different statistical estimation methods.^7^, ^8^, ^9^ The estimates from these methods are roughly in agreement with each other and these estimates suggest that *R*_0_ of MERS‐CoV in the ROK outbreak is quite high with mean of around 2.5‐7.2. Our dynamics models indicate that the transmission process experiences an effectively breaking point at 28 or 29 May 2015 in contact and diagnosis rates. In view of the intervention timeline shown in Figure 1, this effective breaking point indicates that both quarantining close contacts and informing the public the actual situation of outbreak could be the main countermeasures. Before this breaking point, *R*_0_ could range from 5.4 to 9.1 depending on the day of the breaking point. These estimates well overlap with the statistical estimates. Although being significantly higher than the estimates based on the previous small outbreaks, our estimates are compatible with others on the ROK outbreak^16^, ^17^, ^18^ and also the estimate on the 2014 outbreak in the KSA.^14^, ^34^ In view of these analyses, *R*_0_ of MERS‐CoV before the effective intervention can be much higher than the threshold level in the healthcare‐associated environment. This result suggests that MERS‐CoV is not only severe but also highly contagious at least under the Korean healthcare culture.^21^

It is well known that the overall transmissibility of MERS‐CoV in community is very low.^35^ Most introductions are not followed by human‐to‐human transmission or with only limited transmission.^36^ Occasionally due to a range of factors such as long delay from symptom onset to isolation, long stay in hospitals and visiting more healthcare facilities,^37^ more extensive outbreaks can happen such as the spring 2014 outbreak in the KSA.^14^ Naturally if you measure *R*_0_ in one of these larger outbreaks, it will be bigger—it might be difficult to tell whether or not that is only because you have ignored the data from all the individual cases/clusters which suggest much lower *R*_0_. The ROK outbreak is the largest outside of the Middle East and has one clear index case. With all the suspected are closely quarantined and monitored in the ROK,^26^ the chance for missing such individual cases/clusters is extremely low. Therefore, the above results should be robust.

Our dynamics model is simplified in many aspects, such as ignoring the age variation in transmissibility and superspreaders.^27^, ^28^, ^31^ Although transmission was only taken place in hospitals, our model assumes the transmission occurs in the wide population and therefore ignores the setting‐dependent transmission. Understanding how these factors impact the transmission process can provide further information for the outbreak. For example, as far as the non‐pharmaceutical interventions such as closing mass gatherings and school are concerned, it is important to explore the age‐structured population dynamics of transmission. To identify how Korean culture‐associated behaviour affects the spread, it is necessary to investigate the spatial and setting‐dependent transmission dynamics. Even with these limitations, our dynamics model roughly catches the basic characteristics of the transmission dynamics during the ROK outbreak as evidenced by the agreement of its *R*_0_ estimate with statistical methods and reasonable identification of breaking point in contact and diagnosis rates. Hence, it should provide useful information for us to understand the outbreak.

Our estimate of *R*_0_ is different from the previous,^10^, ^11^, ^12^ which appears understandable. For small outbreaks or sporadic cases on which the previous analysis mostly based,^13^ the transmission processes end naturally without intervention**.** The ROK outbreak differed from the previous outbreaks in two ways: it was the largest outbreak outside of the Middle East so far and ended under the strong countermeasures.^21^ Analyses indicate that transmission appears to be amplified in healthcare setting.^10^, ^21^, ^25^, ^27^, ^28^, ^37^Specifically, Chowell et al.^12^ who modelled the progression of MERS‐CoV cases in 2013 concluded that the relative contribution of hospital‐based transmission is over four times higher than that of community transmission. This suggests another reason why the transmissibility of MERS‐CoV in the ROK is much higher than the previous analyses because all the transmission events during the ROK outbreak were observed to occur in hospitals. This in return lends evidence that the healthcare environment is fertile soil for the transmission of MERS‐CoV^14^ and hints the importance of strengthening hospital infection control programmes.

The occurrence of the ROK outbreak also reflects the effect of the Korean traditional culture that relatives and friends would like to visit patients in hospitals and patients have freedom to choose “better” hospitals.^21^ This culture and mobility allow infected people to move among hospitals, and increase the contact rates between the infected and the susceptible; at the early stage of the outbreak, this helps the infection quickly spread. This further hints the reason why when MERS‐CoV hit Westernised countries only limited transmission was experienced, but only causes a big outbreak in the ROK.

Our dynamics modelling shows that facilitating early diagnosis by shortening the delay from symptom onset to confirmation and implementation of infection control measures such as self‐protection and quarantining are efficient measures. Both reflect changes in people's behaviour upon knowing the outbreak situation. The first factor implies quick response and confirmation of the infected people so that the period they can transmit was decreased. The second factor reduces contacts, implying that keeping the community informed the true outbreak situation is important for control. The two interventions (self‐protection and quick diagnosis) might be accomplished by different people: the latter can only be accomplished by those who became ill and their close contacts while everyone in the system can change their behaviours to become self‐protected. In reality, it is likely that these two effects initiated in separate moments; however, the separate breaking points may not much impact the results as indicated in Table 3: the model variant that assumes only time‐varying self‐protection performs nearly as good as the best model that assumes time variation in both self‐protection and diagnosis rate. Thus, using a simultaneous variation in the two interventions should provide a good approximation to the true situation.

Model simulations (data not shown) suggest that if the public awareness of the outbreak could be made 3 days earlier, the total infected people reduced to fewer than 100. However, the simulations also indicate that if the countermeasures were delayed by 3 days, the outbreak size would double. Therefore, the lesson we learned from the outbreak is to rapidly isolate any suspected infected people who are hospitalised and their close contacts.

Lee et al.^28^ also proposed a dynamic transmission model to analyse the MERS‐CoV outbreak in the ROK by explicitly incorporating superspreading events and time‐dependent transmission and isolation rate. As they separated the two largest spread spreaders (of *R *= 80 and 24, respectively) from the whole system, the reproductive number during pre‐intervention was estimated to be below the threshold level of 1.0. Nevertheless, it is interesting to notice the followings. Lee et al.^28^ based on survey^22^ to assume the date that intervention started was 7 June 2015, and the duration from illness onset to diagnosis reduced from 6 days to 2 days. The inference from our transmission dynamics suggested that the objective intervention started from 28 May 2015, and the delay from symptom onset to confirmation decreased from 9.3 to 4.1 days. Lee et al.^28^ also assessed the effect of timing of control measures and like ours found that the early initiation of countermeasures can substantially reduce the size and duration of outbreak.

A historical analysis^38^ indicates the average trend that more than two new species of human virus are reported each year, which was further confirmed by the recent emergence of Swine flu, MERS‐CoV, Ebola and Zika. Anticipation of novel virus species must be included in public health planning. Although we cannot predict their severity and transmissibility, learning from the past outbreaks and thus training our public health service system is an important part of our preparedness. One key lesson learned from the past outbreaks of emerging pathogens including SARS, Ebola and MERS‐CoV is the setting‐dependent transmissibility. For any potentially oncoming novel pathogens, it is hard (if possible at all) to predict their biological properties. The only strategy that can use as countermeasures is to reduce the contact rates such as closing mass gatherings and avoiding any unnecessary contacts. Hence, the knowledge is crucial about what changes in human behaviour and contact patterns help reduce the chance of their emergence and outbreak, or can avoid their rapid and large‐scale diffusion among populations. Nevertheless, hospitals are clearly important settings for amplifying transmissibility,^37^ many of which cases will be hospitalised, thus increasing the risk of exposing healthcare workers and other patients. This highlights the importance of ensuring the early implementation of adequate infection control measures around severe cases to minimise such onward transmission.

## 6 CONCLUSION

Our transmission dynamics model identifies a breaking point at 28 or 29 May 2015 in contact and diagnosis rates. This breaking point reflects the consequence of changes in people's behaviour for self‐protection and Korean authority's designated facilities to isolate and quarantine the close contacts. Our estimates of the reproductive number of MERS‐CoV before the breaking point are in agreement with others on the ROK outbreak, albeit significantly higher than estimates based on other small outbreaks and sporadic cases of MERS‐CoV. The large‐scale outbreak in the ROK was jointly due to the high transmissibility in the healthcare‐associated setting and the Korean culture‐associated contact behaviour. Limiting such behaviour by rapidly identifying and isolating cases and avoiding high‐risk contacts effectively stopped further transmission.

## COMPETING INTERESTS

The authors declare that they have no competing interests.

## AUTHORS’ CONTRIBUTIONS

X‐SZ conceived of the study through discussion with YHC, RP, AC, DDA, PB, HK and MB. X‐SZ carried out the modelling and statistics analyses. X‐SZ, YHC and RP wrote the manuscript. All authors read and approved the final version of the article.

## NOTE

1 For any outbreak of infectious disease that originated from one index case and self‐limited or stopped under control, its overall reproductive number throughout the entire outbreak course has a mean equal to (n − 1)/n for an outbreak of size n. Depending on the structure of the transmission tree, variance in reproductive number can be as small as (n − 1)/n^2^ when each case causes one new case and the last one causes none, and as large as (n − 1)^3^/n^2^ when the index case cause n − 1 secondary cases. Hence the mean reproductive number throughout the outbreak course is of no help for us to understand the transmissibility of the causing pathogens along the whole course of the outbreak.
