# Supplementary material for: Estimating and modelling the transmissibility of Middle East Respiratory Syndrome CoronaVirus during the 2015 outbreak in the Republic of Korea
Source: Influenza Other Respir Viruses. 2017 Aug 17;11(5):434–44. doi: 10.1111/irv.12467 (PMC5598245; doi:10.1111/irv.12467)
Supplement: Supplementary file 2 [file IRV-11-434-s002.docx]

**Supplementary web appendixes**

Supplement to: Xu-Sheng Zhang, Richard Pebody, Andre Charlett, et al. Estimating and Modelling the Transmissibility of Middle East Respiratory Syndrome Corona Virus during the 2015 outbreak in the Republic of Korea

**Appendix A Estimation of basic parameters within transmission dynamics from confirmed cases data**

Two different distributions (negative binomial and gamma) are used to fit the recorded data of the five stage durations: incubation period (Figure A1), delay from symptom onset to confirmation (Figure A2), and serial interval (Figure A3).


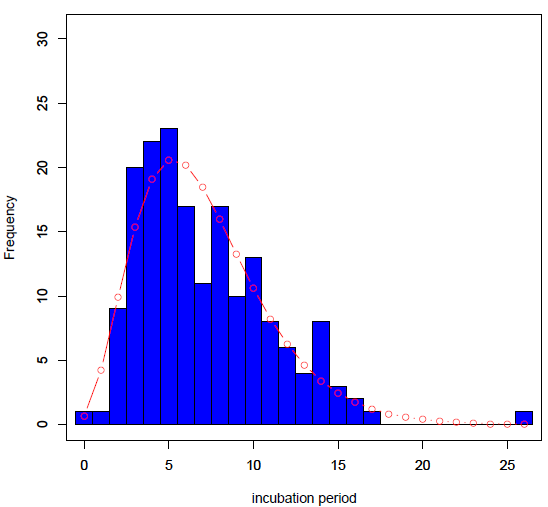


Figure A1 Incubation period (delay from exposed to symptom onset) (Latent period =delay from infected to infectious) (*L*). The best fitted gamma distribution is Γ(4·44, 0·55).


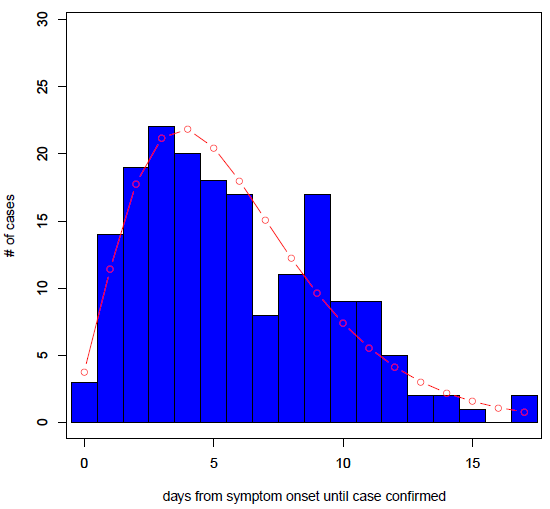


Figure A2 Delay from symptom onset to confirmation (*D*). The delay decreased along with e course of outbreak, indicating the people’s response to the epidemic. The best fitted gamma distribution is Γ(3·28, 0·48).

Variation in delay from symptom onset to confirmation among stages along the course of outbreak:

| Period | Shape parameter | Rate parameter | Mean (days) | SD (days) |
| --- | --- | --- | --- | --- |
| Up to May 28 (breaking point) | 3·76 | 0·41 | 9·17 | 4·73 |
| after May 28  (breaking point) | 3·44 | 0·54 | 6·37 | 3·43 |
| The whole outbreak | 3·28 | 0·48 | 6·83 | 3·77 |


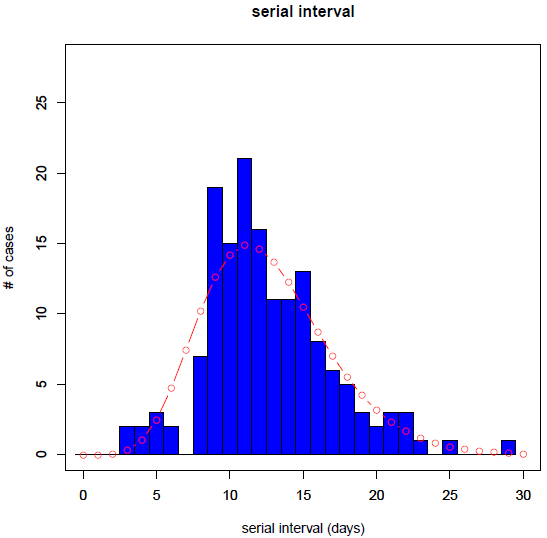


Figure A3 Serial interval distribution. The best fitted gamma distribution is Γ(9·83, 0·72).

**Appendix B: Estimates of reproductive number using package “*R*_0_“**

The reproductive number *R*_t_ at day *t* from package “*R*_0_” (Obadia et al 2012) is estimated by setting the analysis interval from day 1 (symptom onset of the index case) to day *t*. For intervals from day 1 to day *t* with *t* <15, both “EG” and “ML” methods cannot give meaningful results (cf, Hsieh 2015). To search the best *R*_t_ for the basic productive number (*R*_0_), the *R*_t_ and their deviance *r*^2^ statistics (i.e., correlation coefficient between predicted curve) are produced.

The results from **“**EG**”** (exponential growth rate) method are shown in the left panel of Figure B1. The values of *r*^2^ suggest that the estimation based on the interval from day 1 to day 23 is the best one: the exponential growth rate is 0.175 [0.135, 0.218] and *R*_0_= 7·75 [5·05, 12·11]. Notice that the *r*^2^ are high for the estimates based on intervals from day 1 to day 22 to day 1 day 26, the overall average gives: *R*_0_ = 6·36 [4·25,9·68], and exponential rate ranges from 0·119 to 0·194.

The results from **“**ML**”** (Maximum likelihood) method shown in the right panel of Figure B1 indicate that the estimates using intervals from the period of day 1 to day 22 to period of day 1 to day 26 give good estimation. The average estimate among these intervals are *R*_0_ = 5·89[4·42, 7·66] while the period that generate the highest *r*^2^ gives *R*_0_ = 6·40 [4·76, 8·37].


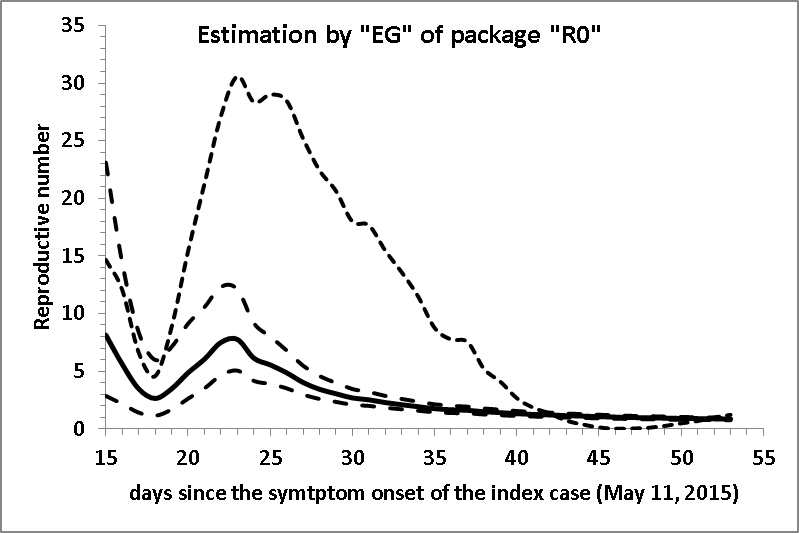

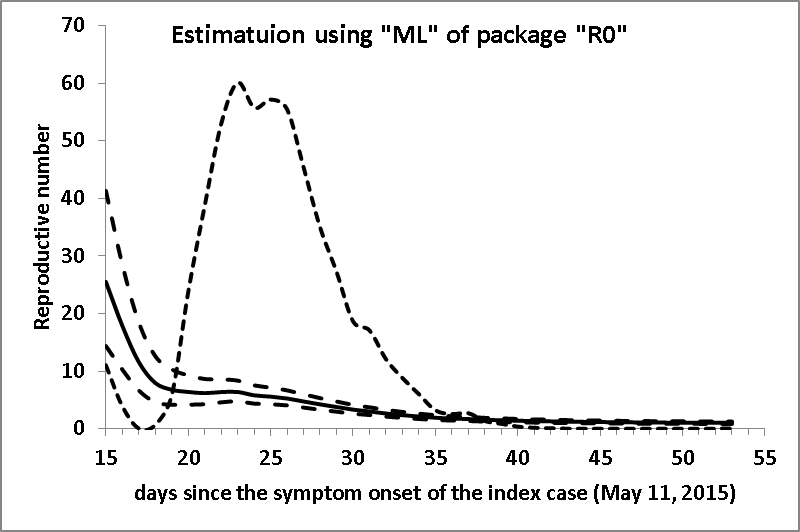


Figure B1 Estimation of reproductive number during the exponential growth phase using package “R0” (Obadia et al 2012). Solid lines represent the median and two dashed lines the 95% credible intervals. The dotted line shows the 50 times (right panel) and 100 times (right panel) the correlation coefficients between predicted curve and observed epidemic curve.

**“**SB**” (**Sequential Bayesian estimation**)** from Obadia et al 2012 This estimate for *R*_t_ includes information on all observations up to time *t* and therefore should be a robust estimator of the effective reproduction number that is assumed to be constant over the whole epidemic up to day *t*. This method approximates the SIR model whose serial interval is roughly the infectious period (Bettencourt and Ribeiro 2008; Obadia et al 2012). Time units less than 5 days lead to time periods of 0 observations at early stage and this fails the SB method. We choose time unit of 5 days and the result are shown in Figure B2. The estimation up to day 22 (i.e., May 30, 2015, which is near the breaking point May 28, 2015) was used as the *R*_0_ listed in Table 1.


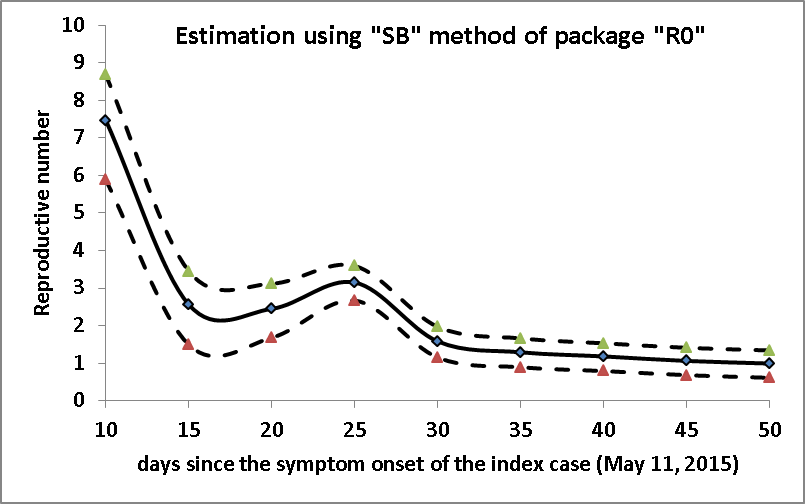


Figure B2. Estimation of reproductive number using “SB” method of package “R0” (Obadia et al 2012). Solid lines represent the median and two dashed lines the 95% credible intervals.

**Appendix C** Results of other reasonable variants of transmission dynamics model

The three other model variants whose results are listed below perform also good in comparison with the best model variant given and discussed in the main text (see Table 1,3, and Figure 2)

Table C1 Posterior medians and 95%credible intervals for parameters under the model assuming the breaking point at May 29 in both contact and diagnosis rates.

| parameter | Mean (SD) | Median (95% CrI) |
| --- | --- | --- |
| Transmission rate (*β*) | 0·98(0·20) | 0·95(0·69,1·46) |
| Incubation period (*L*) | 7·50(1·48) | 7·41(4·87,10·69) |
| Self-protection (*ω*) from May 28 | 0·091(0·039) | 0·084(0·040,0·187) |
| Delay from symptom onset to confirmation (*D*_0_) before May 29 | 7·70(2·98) | 7·76(3·17,14·74) |
| Delay from symptom onset to confirmation (*D*_2_) after May 29 | 4·60(1·30) | 4·50(2·36,7·45) |
| Dispersion parameter (*η*) | 3·87(0·53) | 3·82(2·97,5·05) |
| R_0_ (reproductive number before effective intervention) | 7·15(2·01) | 6·85(4·13,11·92) |
| R_c_ (reproductive number after effective intervention) | 0·365(0·0665) | 0·362(0·245,0·504) |

Table C2 Posterior medians and 95%credible intervals for parameters under the model assuming the breaking point at May 28 in contact rate only.

| parameter | Mean (SD) | Median (95% CrI) |
| --- | --- | --- |
| Transmission rate (*β*) | 1·22(0·22) | 1·19(0·87,1·75) |
| Incubation period (*L*) | 7·97(1·49) | 7·88(5·30,11·18) |
| Self-protection (*ω*) from May 28 | 0·064(0·022) | 0·061(0·032,0·116) |
| Delay from symptom onset to confirmation (*D*_0_) | 5·24(1·45) | 5·11(2·79,8·43) |
| Dispersion parameter (*η*) | 3·87(0·53) | 3·82(2·96,5·05) |
| R_0_ (reproductive number before effective intervention) | 6·17(1·15) | 6·07(4·20,8·70) |
| R_c_ (reproductive number after effective intervention) | 0·376(0·0676) | 0·373(0·252,0·517) |

Table C3 Posterior medians and 95%credible intervals for parameters under the model assuming the breaking point at May 29 in contact rate only.

| parameter | Mean (SD) | Median (95% CrI) |
| --- | --- | --- |
| Transmission rate (*β*) | 1·12(0·22) | 1·09(0·79,1·64) |
| Incubation period (*L*) | 7·37(1·44) | 7·28(4·79,10·44) |
| Self-protection (*ω*) from May 29 | 0·072(0·024) | 0·068(0·036,0·129) |
| Delay from symptom onset to confirmation (*D*_0_) | 5·04(1·41) | 4·92(2·64,8·16) |
| Dispersion parameter (*η*) | 3·89(0·53) | 3·85(3·00,5·06) |
| R_0_(reproductive number before effective intervention) | 5·44(0·98) | 5·35(3·76,7·60) |
| R_c_(reproductive number after effective intervention) | 0·371(0·0683) | 0·368(0·247,0·513) |


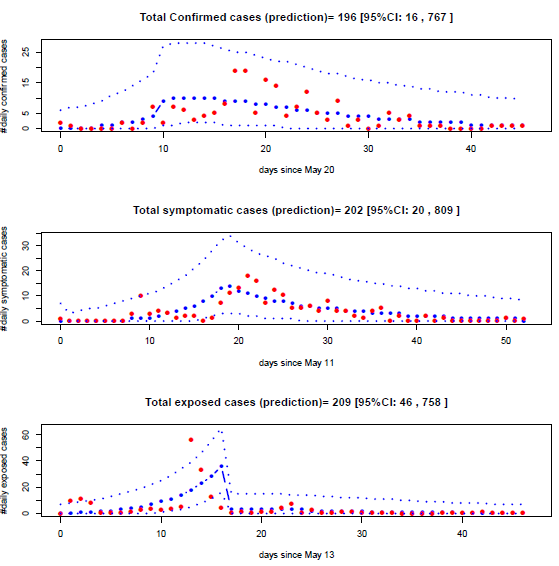


Figure C1 Transmission dynamics model fitting to the confirmed, symptomatic and exposed cases data under Model assuming the breaking point at May 29 in both contact and diagnosis rates. Red filled circles are the cases data and blue circles represent the median predictions from transmission dynamics model and the thin dotted lines represent 95% credible intervals.


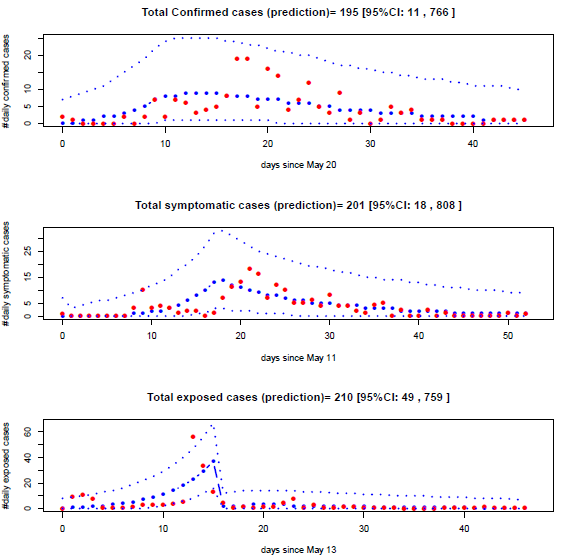


Figure C2 Transmission dynamics model fitting to the confirmed, symptomatic and exposed cases data under Model assuming the breaking point at May 28 in contact rate only. Red filled circles are the cases data and blue circles represent the median predictions from transmission dynamics model and the thin dotted lines represent 95% credible intervals.


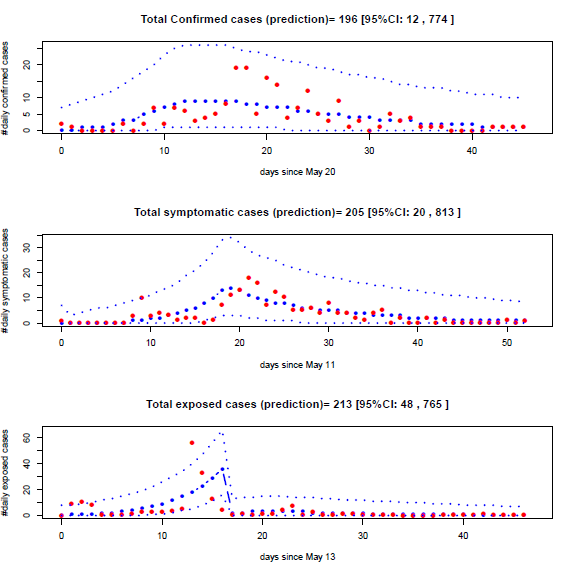


Figure C3 Transmission dynamics model fitting to the confirmed, symptomatic and exposed cases data under Model assuming the breaking point at May 29 in contact rate only. Red filled circles are the cases data and blue circles represent the median predictions from transmission dynamics model and the thin dotted lines represent 95% credible intervals.

**References:**

Bettencourt LMA, Ribeiro RM. Real time Bayesian estimation of the epidemic potential of emerging infectious diseases. PLOS One 2008; 3:e2185 http://dx.doi.org/10.1371/ journal.pone.0002185.

Hsieh Y-H. 2015 Middle East Respiratory Syndrome Coronavirus (MERS-CoV) nosocomial outbreak in South Korea: insights from modeling. PeerJ 2015; 3:e1505; DOI 10.7717/peerj.1505.

Obadia T, Haneef R, Boelle P-Y. The R0 package: a toolbox to estimate reproduction numbers for epidemic outbreaks. BMC Med Inform Decis Mak 2012; 12:147 <http://www.biomedcentral.com/1472-6947/12/147>.
